# Scheimpflug-Derived Corneal Lower and Higher Order Aberrations Post Intrastromal Corneal Ring Segments for Keratoconus

**DOI:** 10.3390/vision6040076

**Published:** 2022-12-14

**Authors:** Roberta M. van den Berg, Arthur B. van den Berg, Maya Dodhia, Michel Shahid, Alessandro A. Jammal, Denise de Freitas, Karolinne M. Rocha

**Affiliations:** 1 Storm Eye Institute, Medical University of South Carolina (MUSC), Charleston, SC 29425, USA; 2 Ophthalmology Department, Federal University of Sao Paulo (UNIFESP), São Paulo 04023-062, Brazil; 3 School of Medicine Columbia, University of South Carolina, Columbia, SC 29208, USA; 4 Duke Eye Center, Duke University, Durham, NC 27708, USA

**Keywords:** keratoconus, intrastromal corneal ring segments, higher-order aberrations, pupil size

## Abstract

Intrastromal corneal ring segments (ICRS) improve corneal topographic symmetry and reduce corneal aberrations through regularization of the corneal surface, thereby functioning as a viable surgical intervention for patients with keratoconus. This study aims to evaluate changes in lower- (LOAs) and higher-order aberrations (HOAs) amongst varying pupil sizes pre- and post- ICRS implantation in keratoconus patients. We specifically investigate the impact of pupil size on total corneal HOAs up to the 6th order. Twenty-one eyes that underwent ICRS implantation were included in this prospective interventional study. LOAs and HOAs measurements at the 6 mm, 4 mm, and 2 mm pupil diameters were collected preoperatively and at 6 months postoperatively using the Zernicke analysis function on a Scheimpflug device. ICRS implantation demonstrated a statistically significant effect in vertical coma with a −0.23 reduction (*p* = 0.015) for a 4 mm pupil size and a −1.384 reduction (*p* < 0.001) for 6 mm, with no significant effect at 2 mm. Horizontal coma, astigmatism 0°, astigmatism 45°, trefoil 5th order 30°, and RMS HOA demonstrated significant reductions at 4 mm or 6 mm pupil sizes but not at 2 mm. Our analysis demonstrates a favorable effect of ICRS implantation on larger pupil sizes, suggesting the importance of pupil size as it correlates with HOAs reduction.

## 1. Introduction

Intrastromal corneal ring segments (ICRS) are FDA-approved medical devices under a Humanitarian Device Exemption (HDE), implanted within the corneal stroma to change cone geometry [1]. ICRS were initially designed to correct myopia in normal eyes [2] and are also indicated to minimize spherocylindrical errors in patients with keratoconus, who can no longer achieve adequate functional vision with their contact lenses or spectacles [3]. These hexagonal-shaped 150° polymethyl methacrylate (PMMA) arcs are inserted between deep stromal layers of the cornea to improve corneal topographic symmetry.

The implantation of ICRS is a reversible procedure and an alternative treatment that can delay corneal transplantation [4]. ICRS are also used to reduce corneal higher-order aberrations (HOAs) and astigmatism through the regularization of the corneal surface [3,5]. Previous studies have shown improvement in an overall vertical coma and total corneal HOAs after ICRS implantation [6]. While there is evidence that smaller pupils are less influenced by aberrations [7], the effect of ICRS in different pupil sizes has not been fully evaluated.

This study aims to evaluate changes in corneal lower and higher-order aberrations using different pupil sizes before and after the implantation of ICRS using a Scheimpflug device. In addition, we investigated potential associations among a simulated range of mesopic and photopic pupil diameters on visual function and quality of vision as measured by total corneal wavefront aberrations. To the best of our knowledge, this is the first study to investigate the impact of pupil size on total corneal HOAs up to the 6th order pre- and post-implantation of ICRS.

## 2. Materials and Methods

### 2.1. Clinical Population

This prospective study included patients who underwent intrastromal corneal ring segments implantation at the Storm Eye Institute, Medical University of South Carolina (MUSC), from July 2016 to April 2021. The study protocol was approved by the MUSC Institutional Review Board (IRB). Informed consent was obtained from all subjects prior to ICRS implantation. All methods adhere to the tenets of the Declaration of Helsinki. 

Keratoconus patients 21 years or older presenting for ICRS implantation with inferior or central corneal steepening, corrected distance visual acuity (CDVA) worse than 20/20 (0.0 logMAR), and history of contact lens intolerance, were included in the study. Subjects with a history of corneal disorders other than keratoconus at presentation (e.g., corneal dystrophies, ocular surface disease, ocular trauma, herpetic keratitis, corneal melt, recurrent corneal erosion, corneal scarring), nystagmus, and/or corneal pachymetry measurements lower than 400 µm at the thinnest point, or if pregnant or lactating at the time of the study were excluded.

### 2.2. Surgical Technique

The surgical technique consisted of creating a corneal tunnel under topical anesthesia using the femtosecond laser (FS200, Alcon, Forth Worth, TX, USA), which performs a continuous circular 8 mm outer diameter and 6.8 inner diameter tunnel with 75% corneal depth within 7 s. After the femtosecond laser treatment, the ICRS (Intacs, AJL Ophthalmic S.A., Spain) were implanted. All procedures were performed by the same experienced surgeon (KMR).

### 2.3. Imaging

Scheimpflug imaging with the Pentacam HR^®^ (Oculus GmbH, Wetzlar, Germany) was performed pre-and post-operatively. Refractive outcomes, including manifest refraction, spherical equivalent (SE), and CDVA were collected from clinical visits from electronic medical records before and after surgery. Corneal asphericity (Q value), keratometry values (K1 and K2), mean keratometry (Km), and maximum keratometry (Kmax) were extracted from the “4 Maps Refractive” report of the Pentacam HR^®^. Lower-order aberrations (LOAs) and HOAs were obtained from the Zernike analysis report map. The Pentacam HR automatically converts the corneal elevation profile into corneal wavefront data for different pupil sizes using the Zernike polynomials. The investigators predetermined the 6 mm, 4 mm, and 2 mm pupil diameters to simulate a range of mesopic and photopic light conditions for all participants. Post-operative measurements were performed 6 months after the procedure.

### 2.4. Statistical Analysis

Normality was checked using the Shapiro–Wilk test and the Kolmogorov-Smirmov test. One sample t-test was used to assess differences pre- and post-intervention if variables were normally distributed. The Wilcoxon rank-sum test was used if one or more variables were not normally distributed. To evaluate the effect of the intervention in each variable of interest, generalized least squares (GLS) population-averaged linear models with random-effects analysis were applied for aberrations for pupil sizes 6 mm, 4 mm, and 2 mm. For the regression models, only the absolute value for each parameter was considered to indicate the intervention’s effect on the magnitude of the aberrations at each pupil size. Statistical analysis was performed using Stata version 17.0 (Stata Corp LLC, College Station, TX, USA). A *p* value < 0.05 was considered statistically significant.

## 3. Results

The study included 21 eyes (18 individuals) with a mean age of 37.7 ± 9.6 years, and 12 (57.14%) subjects were female. There were no intra or postoperative complications.

At baseline, mean± standard deviation (SD) CDVA was 0.52 ± 0.31 logMAR and spherical equivalent was −3.56 ± 3.95 D. Mean± SD CDVA improved to 0.44 ± 0.49 logMAR (*p* = 0.521) and spherical equivalent reduced to −2.09 ± 5.35 D (*p* = 0.329) at the 6-month postoperative visit, with a reduction of Kmax from 66.40 ± 12.48 D to 60.90 ± 8.06 D (*p* = 0.098). The distributions of the refractive outcomes pre- and post-intervention are shown in Table 1.

Univariable regression models were used to assess the effect of ICRS implantation in keratoconus and included in this study (Table 2). Using univariable regression models, Kmax showed statistically significant improvement after ICRS with a −5.49 D reduction (95% CI: −10.30, −0.69 D; *p* = 0.025).

**Figure 1:**
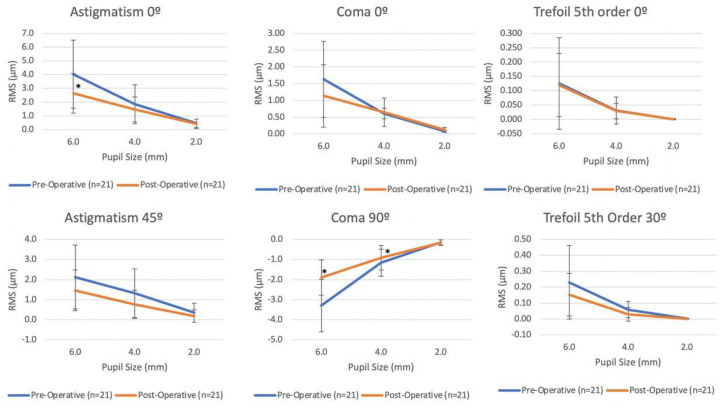
Pre- and post-operative absolute mean values in root mean square (RMS) higher- (HOA) and lower-order aberration (LOA) at 6 mm, 4 mm, and 2 mm pupil diameters. Comparison of pre-operative and post-operative measurements of Astigmatism 0°, Coma 0°, Trefoil 5th Order 0°, Astigmatism 45°, Coma 90°, and Trefoil 5th Order 45° at pupil sizes of 6.0 mm, 4.0 mm, and 2.0 mm. Error bars indicate standard deviation. * Results were found to be statistically significant *p* < 0.05. Statistical analysis was obtained using generalized least squares linear models comparing pre-operative and post-operative measurements at each pupil size.

Overall, the impact of the intervention was greater the larger the pupil size (Figure 1). The intervention showed a statistically significant effect in vertical coma with a 0.23 absolute reduction (95% CI: −0.417 to −0.044; *p* = 0.015) for a 4 mm pupil size and a 1.384 absolute reduction (95% CI: −1.822 to −0.945; *p* < 0.001) for 6 mm, whereas no significant change was seen for pupils of 2 mm in diameter (0.055; 95% CI: −0.031 to 0.041; *p* = 0.802). Similarly, horizontal coma, astigmatism 0°, astigmatism 45°, trefoil 5th order 30°, and RMS HOAs demonstrated significant reductions at 4- or 6-mm pupil sizes, but not at 2 mm. None of the variants had a statistically significant reduction in a 2 mm pupil. (Figure 1).

Of note, even when a significant change in the aberration was observed for pupil sizes of 2 mm-diameter, the magnitude of the change was rather small and likely not clinically relevant. For example, values for pentafoil 18°, hexafoil 15°, and horizontal coma 5th order showed a significant increase at 2 mm, but such effect was minimal (0.002, 95% CI: 0.001 to 0.002, *p* < 0.001; <0.001, 95% CI: 0.000 to 0.001, *p* < 0.001; 0.001, 95% CI: 0.000 to 0.002, *p* < 0001, respectively), while at 6 mm the same parameters demonstrated a much greater magnitude of change, with an average increase of 0.156 (95% CI: 0.073 to 0.239, *p* < 0.001), 0.082 (95% CI: 0.020 to 0.143, *p* = 0.009), and 0.184 (95% CI: 0.100 to 0.268, *p* < 0.001, respectively.

**Figure 2:**
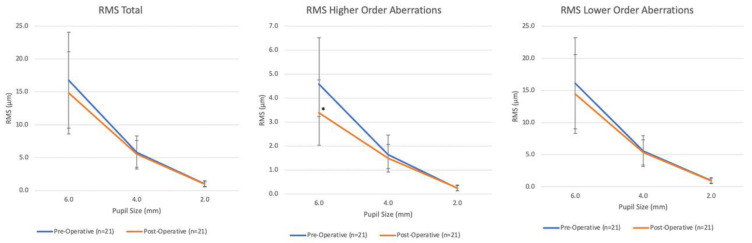
Pre- and post-operative absolute mean values in root mean square (RMS) higher- (HOA) and lower-order aberration (LOA) at 6 mm, 4 mm, and 2 mm pupil diameters. Comparison of pre-operative and post-operative measurements of total aberrations, higher order aberrations, and lower order aberrations at pupil sizes of 6.0 mm, 4.0 mm, and 2.0 mm. Error bars indicate standard deviation. * Results were found to be statistically significant *p* < 0.05. Statistical analysis was obtained using generalized least squares linear models comparing pre-operative and post-operative measurements at each pupil size.

We observed no statistically significant improvement after surgery in RMS LOAs and RMS total predictions in all pupil sizes from the Scheimpflug Zernike analysis (Figure 2). However, there was a significant reduction in RMS HOAs of −1.189 (95% CI: −1.775, −0.603; *p* < 0.001), which was not observed at 2- or 4-mm pupil size (*p* = 0.645 and *p* = 0.149, respectively) Postoperatively, there was no significant worsening of the spherical aberration (SA) and vertical coma 5th order parameters for all pupil sizes.

## 4. Discussion

Keratoconus can be defined by a progressive weakening and thinning of the cornea, which results in irregular astigmatism and decreased quality of vision [8,9]. Increased corneal HOAs are optical consequences of keratoconus and corneal ectasia [10]. ICRS have been described to enhance corneal optical symmetry and topography in keratoconus [5,11,12]. By improving the corneal optical parameters, HOAs would be expected to be decreased. Although ICRS’s effect in reducing ectatic progression is questionable, this procedure is indicated to improve visual acuity and function. 

Previous studies reported the effectiveness of this procedure in reducing the spherical equivalent and keratometric measures due to central cornea flattening in keratoconus patients [13,14,15,16,17]. The authors also described improvement in uncorrected and corrected visual acuities, SE, and cylinder. Benoist d’Azy et al. [18] published a systematic review and meta-analysis evaluating the efficacy of the keratometric and refractive outcomes of ICRS inserts in 2019. The analysis included 64 studies, and the authors concluded that the procedure significantly improved all assessed parameters (UDVA, CDVA, Kmax, and Kmean). Our findings are in accordance with the literature: we observed a statistically significant flattening in Kmax (−5.49 D; −95% CI: −10.30, −0.69 D; *p* = 0.025) and Kmean (−3.90 D; 95% CI: −6.34, −1.47 D; *p* = 0.002). CDVA also had a slight improvement of −0.09 logMAR (95% CI: −0.27 to 0.09 logMAR; *p* = 0.347), but this change was not statistically significant in regression models.

In addition, prior studies described the effect of ICRS implantation on optical quality by analyzing changes in anterior corneal HOAs [6]. The authors have found a reduction in these parameters, particularly in the asymmetric aberrations (coma and coma-like). Of note, none of these studies correlated the pupil sizes and anterior corneal aberrations. These modifications detected in the Zernike coefficients report are expected due to the implants’ potential to regularize the corneal tissue’s geometry [6,19,20]. In our study, at 6 months post-ICRS implantation, corneal anterior LOAs and HOAs, especially vertical coma, trefoil 5th order 30°, and astigmatism 45°, improved significantly for 4- and 6-mm pupil sizes. There was also a statistically significant reduction of anterior corneal HOAs −1.189 (95% CI: −1.775 to −0.603; *p* < 0.001) for 6 mm (Figure 2).

Vertical coma (anterior corneal HOA) is considered an important aberration in keratoconus [6], consequently, the improvement of this parameter postoperatively can be particularly favorable. As reported by Greenstein et al. [21], the vertical coma had the most significant improvement, however, the pupil size analyzed was not described. In our study, the vertical coma reduced −1.384 (95% CI: −1.822 to −0.945; *p* < 0.001) compared to the absolute pre-operative value for a 6 mm pupil size and −0.230 (95% CI: −0.417 to −0.044; *p* = 0.015) for 4 mm, whereas at 2 mm pupil size, this change was of small magnitude and not significant (0.005; 95%CI: −0.031, 0.041; *p* = 0.802). These findings highlight that, although an intervention with ICRS can be effective in reducing aberrations in keratoconus, pupil size plays an important role in patient outcomes, with a favorable effect of ICRS implantation on larger pupil sizes. Negative spherical aberration has been reported in patients with keratoconus due to steepening of the cornea and its hyperprolate shape [21]. In our study, the absolute value of SA increased, on average, for all pupil sizes (0.011; 95% CI: −0.01 to 0.024; *p* = 0.064 for 2 mm, 0.108; 95% CI: −0.051, 0.266 *p* = 0.182 for a 4 mm pupil and 0.094; 95% CI: −0.549 to 0.737; *p* = 0.775 for 6 mm), although the effect was not statistically significant. This increase in the SA profile may explain why the *coma* decreased by more than the total anterior corneal HOAs after surgery. The etiology of this mild increase of SA might be due to the more oblate shape of the cornea because of the ICRS flattening effect. 

The pupil size is an important parameter to be considered in wavefront maps analysis [7] and, the impact of peripheral aberrant light rays is minimized according to its reduction, improving visual acuity since no significant differences were found with a 2 mm pupil diameter.

The correlation between pupil size and total corneal wavefront aberrations found in this study indicates that post-ICRS keratoconus patients may benefit from small aperture devices as demonstrated by statistically significant improvement in lower and higher order aberrations. This can help clinical practice guidelines in which the pupil size of post-ICRS patients can be artificially modified to reduce the impact of corneal aberrations to improve visual function. The pinhole effect, utilized by small aperture devices, including small aperture IOLs and pharmacological agents, may improve the quality of vision in patients with aberrated corneas [22].

This study has limitations. The effect on different pupil sizes was predicted based on proprietary algorithms from the device and the natural pupil size in each patient was not considered in our study. Higher-order aberrations are pupil size dependent, and the measurements needed to be standardized for comparison. However, our analysis can help estimate the effect of the aberrations in different light conditions. Additionally, patient-reported quality of life outcomes questionnaires can be used in future studies to assess the impact of small aperture optics on the subjects’ visual function.

## 5. Conclusions

In accordance with previous literature, this study demonstrated an improvement in refractive outcomes and Scheimpflug-based metrics after ICRS implantation. Our findings also suggest that significant improvements in HOAs post-ICRS placement are observed at larger pupillary diameters of 4 and 6 mm, but the change is small at pupil sizes of 2 mm in diameter. Our analysis contributed to the knowledge of the behavior of HOAs in different pupil sizes in highly aberrated eyes after ICRS surgery.

## Figures and Tables

**Table 1:** Refractive outcomes pre- and post-intrastromal corneal ring segments implantation.

	Pre Op (*n* = 21)	Post Op (*n* = 21)	*p* Value
Manifest Refraction Spherical Equivalent (SE) (D)	−3.56 ± 3.95	−2.09 ± 5.35	0.329 ^a^
Cylinder (D)	3.51 ± 2.48	2.65 ± 2.01	0.235 ^b^
Corrected Distance Visual Acuity (CDVA) (logMAR)	053 ± 0.31	0.44 ± 0.49	0.521 ^b^
Mean Keratometry (KM) (D)	54.11 ± 7.78	50.21 ± 5.36	0.066 ^b^
Maximum Keratometry (Kmax) (D)	66.40 ± 12.48	60.91 ± 8.06	0.098 ^b^
Corneal asphericity (Q value)	−1.23 ± 0.67	−1.07 ± 0.68	0.448 ^a^

All values are shown as mean ± standard deviation, unless otherwise noted. ^a^ One sample t-test; ^b^ Wilcoxon rank-sum test.

**Table 2:** Intrastromal corneal ring segments effect on different pupil sizes. Differences pre- and post-operative were calculated from absolute values. Negative coefficients indicate reduction for a given parameter after surgery.

	Pupil Size *		
Parameter	2 mm Pupil (*n* = 21)	4 mm Pupil (*n* = 21)	6 mm Pupil (*n* = 21)
Defocus (µm)	−0.029 (−0.064, 0.005) *p* = 0.095	−0.483 (−1.166, 0.199) *p* = 0.165	−1.794 (−4.548, 0.961) *p* = 0.202
Astigmatism 0° (µm)	−0.007 (−0.165, 0.152) *p* = 0.933	−0.352(−0.993, 0.289) *p* = 0.281	−1.347 (−2.430, −2.630) ***p* = 0.015**
Astigmatism 45° (µm)	−0.109 (−0.286, 0.069) *p* = 0.230	−0.506 (−0.952, −0.600) ***p* = 0.026**	−0.663 (−1.192, −0.135) ***p* = 0.014**
Coma 0° (µm)	0.034 (0.007, 0.062) *p* = 0.014	.036 (−0.078, 0.149) *p* = 0.539	−0.494 (−0.808, −0.180) ***p* = 0.002**
Coma 90° (µm)	0.005 (−0.031, 0.041) *p* = 0.802	−0.230 (−0.417, −0.044) ***p* = 0.0015**	−1.384 (−1.822, −0.945) ***p* < 0.001**
Trefoil 0° (µm)	−0.003 (−0.025, 0.019) *p* = 0.810	−0.013 (−0.118, 0.091) *p* = 0.803	0.034 (−0.097, 0.165) *p* = 0.611
Trefoil 30° (µm)	−0.007 (−0.028, 0.015) *p* = 0.539	0.011 (−0.155, 0.178) *p* = 0.893	−0.034 (−0.399, 0.330) *p* = 0.853
Astigmatism 4th order 0° (µm)	0.007 (−0.004, 0.017) *p* = 0.221	0.042 (−0.019, 0.103) *p* = 0.174	0.098 (−0.118, 0.313) *p* = 0.374
Astigmatism 4th order 45° (µm)	0.001 (−0.009, 0.010) *p* = 0.913	−0.021 (−0.121, 0.078) *p* = 0.676	−0.232 (−0.492, 0.028) *p* = 0.080
Spherical Aberration (µm)	−0.011 (−0.001, 0.240) *p* = 0.064	0.108 (−0.051, 0.266) *p* = 0.182	0.094 (−0.549, 0.737) *p* = 0.775
Pentafoil 18° (µm)	0.002 (0.001, 0.002) ***p* < 0.001**	0.037 (0.017, 0.057) ***p* < 0.001**	0.156 (0.073, 0.239) ***p* < 0.001**
Coma 5th order 0° (µm)	0.001 (0.000, 0.002) ***p* = 0.025**	0.041 (0.018, 0.065) ***p* = 0.001**	0.184 (0.100, 0.268) ***p* < 0.001**
Spherical Aberration 6th order (µm)	<0.001 (0.000, 0.001) *p* = 0.379	0.010 (−0.004, 0.024) *p* = 0.180	0.123 (0.048, 0.199) ***p* = 0.001**
RMS LOA (µm)	−0.041 (−0.205, 0.124) *p* = 0.626	−0.210 (−0.982, 0.562) *p* = 0.593	−1.679 (−4.013, 0.655) *p* = 0.159
RMS HOA (µm)	0.011 (−0.037, 0.059) *p* = 0.645	−0.153 (−0.387, 0.081) *p* = 0.199	−1.189 (−1.775, −0.603) ***p* < 0.001**
RMS Total (µm)	−0.039 (−0.208, 0.130) *p* = 0.650	−0.246 (−1.048, 0.556) *p* = 0.547	−1.925 (−4.309, 0.459) *p* = 0.113

* Univariable GLS (95% CI, *p* value); boldface indicates statistical significance (*p* < 0.05). RMS = root mean square; LOA = lower-order aberration; HOA = higher-order aberration.

## Data Availability

The datasets generated during and/or analyzed during the current study are available from the corresponding author on reasonable request.
